# Association between the use of social networking sites, perceived social support, and life satisfaction: Evidence from a population-based survey in Japan

**DOI:** 10.1371/journal.pone.0244199

**Published:** 2020-12-18

**Authors:** Takashi Oshio, Hiromi Kimura, Toshimi Nishizaki, Takashi Omori

**Affiliations:** 1 Institute of Economic Research, Hitotsubashi University, Kunitachi, Japan; 2 Survey Research Center, Chuo-ku, Japan; 3 Japan Cabinet Office, Chiyoda-ku, Japan; 4 Osaka University, Suita, Japan; Uniwersytet Jagiellonski w Krakowie Biblioteka Jagiellonska, POLAND

## Abstract

This study examined the association between the use of social networking sites (SNS), perceived social support (PSS), and life satisfaction (LS), focusing on the mediating effect of PSS on the association between SNS use and LS. To this end, we used data (*N* = 15,574) obtained from a population-based, nationwide internet survey conducted in Japan. First, we confirmed that the number of SNS friends was positively associated with life satisfaction for all age groups: young (15–29 years), middle-aged (30–59 years), and old (60–86 years). However, the association was mixed if there were 100 or more SNS friends. Second, our structural equation modeling analysis underscored the mediating effect of PSS on the association between the number of SNS friends and LS for all age groups. Specifically, PSS mediated 36.5% (standard error [SE]: 8.6%), 39.8% (SE: 6.3%), and 40.3% (SE: 11.4%) of the association for the young, middle-aged, and old groups, respectively, if we defined SNS use as having 10 or more SNS friends. The mediating effect of PSS consistently contributed to the positive association between SNS use and LS regardless of the number of SNS friends, suggesting that SNS use has the potential to enhance subjective well-being via its positive impact on PSS.

## Introduction

The use of social networking sites (SNS), such as Facebook, LINE, and Twitter, has become increasingly popular not only among the younger and older generations. Widespread SNS use is changing how individuals engage with others [1], inspiring researchers to examine whether SNS use can have a favorable impact on the subjective well-being (SWB) of SNS users. Results obtained from recent relevant studies are generally inconsistent and mixed [2–6]. Although some studies have found SNS use to favorably impact various aspects of SWB, such as life satisfaction (LS) [7–9], strong skeptical views concerning the positive impact of SNS use on SWB exist as well [10, 11].

One possible reason for this inconsistency could be that most previous studies were based on relatively small samples (often well below 1,000), mainly consisting of college students or other young individuals. This probably caused the results to be affected by the group-specific attributes, thereby reducing their statistical reliability. The estimation results may also depend on the research design. For example, comparing SNS users and non-users as well as focusing on the correlations of frequency, intensity, or the number of friends within SNS users alone may lead to results that present different evaluations concerning the impact of SNS use. In addition, many studies mainly focused only on the use of Facebook.

The association between SNS use and SWB may also differ between age groups [12, 13]. There must be substantial differences in frequency, intensity, and contextual meaning of SNS use by age group, probably making the relevance of SNS use differ between the young and old. Studies focusing on older SNS users generally support its beneficial impact on SWB [13–15], but the relative importance of SNS use for SWB between age groups has been largely understudied.

Inconsistent results regarding the association between SNS use and LS have made it increasingly important to explore the mechanism linking the two. In this respect, perceived social support (PSS) has attracted increasing attention, referring to the perceived availability and adequacy of social connections, whereas received social support focuses on the quantity and quality of the support provided when needed [16, 17]. It is reasonable to expect that individuals who perceive a sufficient level of social support may believe that their network will provide adequate help when needed, and thus feel more satisfied. Indeed, studies have shown that PSS can improve SWB and mental health more consistently than received social support [16, 18, 19]. Social networking is expected to provide more opportunities to obtain PSS through greater interpersonal interactions in the online environment [20]. This view is consistent with the finding that acquiring social support from others was one of the most important reasons for SNS use [21]. It should be noted, however, that some studies were skeptical of the positive impact of SNS use on PSS [22].

If PSS is obtained by SNS use and in turn enhances SWB, we can argue that PSS acts as an important mediator for their association. Several studies have provided evidence in support of this view [6, 8, 9], even though its effect was found to be non-linear [23], dependent on personal traits [24], and limited to some of its aspects [25]. However, few studies have evaluated the relative importance of the mediating effect of PSS on the total association between SNS use and SWB.

Following these preceding studies and considering the hitherto unaddressed issues, this study compared the association between SNS use, PSS, and LS (as a proxy of SWB) across three age groups. To this end, we used data obtained from a large-scale population-based nationwide survey conducted in Japan (*N* = 15,574). Survey participants consisted of both SNS users and non-users. In Japan, approximately 80 million persons, i.e., 64% of total population, are estimated to be SNS users and 77.4%, 38.5%, and 35.7% of them use LINE, Twitter, and Instagram, respectively [26].

We mainly focused on the number of SNS friends as a key variable of SNS use and examined its associations with LS and PSS, following preceding studies [8, 9, 23]. To explore the mechanism that links SNS use and LS, we specifically focused on the mediating effect of PSS on this association. Based on the estimation results of the structural equation modeling analysis, we examined whether PSS mediated the association between SNS use and LS and the extent of this impact, comparing the results by age group. We tentatively hypothesized that SNS use may be positively associated with LS and that PSS may mediate a substantial proportion of their association, considering the general results obtained from preceding studies conducted outside Japan. Nevertheless, the ways in which the association among these variables may differ by age group remains largely understudied.

## Materials and methods

### Study sample

We used data obtained from a population-based, nationwide internet survey conducted in the research project of the Cabinet Office (CAO) of the Japanese government in October 2019 and February 2020. The survey was conducted in accordance with the Statistics Law in Japan, which governs the statistical, legal, ethical, and other rules for surveys conducted by the government. Informed consent was obtained from all respondents. We obtained the survey data with permission from the CAO. Hence, ethics approval was not required for this study.

The survey was conducted using the CAO as follows. First, the questionnaires were distributed to the registrants of an Internet survey company. Data were collected from approximately 15,000 respondents: approximately 10,000 from the survey in 2019 and the remaining 5,000 from the survey in 2020. The targeted sample was divided into two groups. First, 11,280 registrants were planned to be allocated equally to each of the 47 prefectures, by gender and age groups (aged 15–24 years, 25–34 years, 35–44 years, 45–59 years, and 60+ years). Thus, each prefecture-gender-age group consisted of 24 individuals. Second, 4,245 registrants were planned to be allocated to each gender-age group in each prefecture, corresponding to its actual population size. When the survey was closed, data for 15,574 respondents were obtained. The dataset is open to the public and available from the CAO website (URL: https://form.cao.go.jp/keizai2/opinion-0011.html).

### Measures

Regarding an indicator of SNS use, we focused on the reported number of SNS friends. The survey asked the respondents to choose from 0, 1–9, 10–19, 20–29, 30–39, 40–49, 50–99, 100–300, and 301 or above. In the regression analysis, we condensed the responses into four categories (0, 1–9, 10–100, 100, and above), corresponding to cutoffs of 1, 10, and 100. Because we set these cutoffs arbitrarily, we additionally conducted a statistical analysis using the number of SNS friends as a continuous variable. In this analysis, we transformed the reported number of SNS friends: 0, 1–9, 10–19, 20–29, 30–39, 40–49, 50–99, 100–300, and 301 or above to 0, 5, 14.5, 24.5, 34.5, 44.5, 74.5, 200, and 400, respectively, taking the mid-point of each band (and arbitrarily transforming “for 301 or above” to 400).

Regarding SWB, we focused on LS on an 11-point scale (0 = *not satisfied at all* and 10 = *highly satisfied*). Regarding PSS, the survey first asked the respondents whether they had anyone they expected to support them when needed (other than their family members and relatives who reside with them). If they answered “yes,” the survey further asked them to report the number of such persons by choosing from among 1, 2, 3, 4, 5, 6–9, 10–19, 20–29, and 30 or more. Based on these reported numbers, we construct a continuous proxy for PSS by allocating 0 to *none* and replacing 10–19, 20–29, and 30 or more, by 14.5, 24.5, and 40, respectively, taking the mid-point of each band (and arbitrarily transforming for “30 or above” to 40).

As for covariates, we considered gender, age group (29 or below, 30s, 40s, 50s, and 60 or above), educational attainment (graduated or scheduled to graduate [in the case of current students] from junior high school, high school, or college or above), job status (regular employee, non-regular employee, manager, self-employed, working part-time from home, student with a job, student with no job, unemployed, and out of labor force), marital status (having a spouse or not), and household income. Household income was adjusted by household size by dividing household income by the square root of the number of household members and then constructed four binary variables for each quartile. We also controlled for the survey years (2019 or 2020).

### Analytic strategy

For the descriptive analysis, we compared the associations of the number of SNS friends with life satisfaction and PSS between age groups. We divided the respondents into three age groups: young (15–29 years), middle-aged (30–59 years), and old (60–86 years), assuming that SNS use and its impact on LS may differ across stages of working life. People are likely to finished entering the labor market by age 30 and start leaving it at age 60 due to mandatory retirement and/or eligibility for public pension benefits in Japan.

We conducted two types of regression analysis. First, we estimated a basic regression model to examine how LS and PSS were each associated with the number of SNS friends. This analysis was aimed at evaluating the results of the descriptive analysis by controlling for the covariates. Second, we conducted a structural equation modeling (SEM) analysis to determine the structural associations between SNS use, LS, and PSS. Based on the SEM analysis, we computed the effects of SNS use on LS, both mediated and not mediated by PSS.

For the first regression analysis, we considered the following linear regression equation (Model 0) in the case of LS:

$$Model\;0:\;LS=\alpha +\beta_{01}SNS_{1+}+\beta_{02}SNS_{10+}+\beta_{03}SNS_{100+}+\left(covariates\right)+\varepsilon_{0},$$

where SNS_1 +_, SNS_10 +_, and SNS_100 +_ are binary variables for the number of SNS friends of 1 or more, 10 or more, and 100 or more, respectively, and *ɛ*_0_ is an error term. We avoided using a continuous variable for the number of SNS friends, considering the possibility of its non-linear relationship with LS.

In this model specification, a positive coefficient of each SNS variable means that LS will increase when the number of SNS friends rises to the corresponding range from the range below. For example, *β*_02_ (i.e., the coefficient of SNS_10+_) > 0 means that LS will increase when the number of SNS friends increases to the range between 10 and 100 from the range between 1 and 9. In this case, we can argue that 10 was a threshold for the number of SNS friends in terms of its association with LS. The coefficients of the conventionally defined binary variables for 1–9, 10–99, and 100+ SNS friends (using no SNS friends as a reference) are equal to *β*_01_, *β*_01_ + *β*_02_, and *β*_01_ + *β*_02_ + *β*_03_, respectively. We estimated Model 0 for PSS as well to examine their relationships with the number of SNS friends.

For the SEM analysis, we considered the structural equation model, which consists of two equations (Models 1 and 2):

$$Model\;1:\;LS=\alpha_{1}+\beta_{1}SNS+\gamma_{1}PSS+\left(covariates\right)+\varepsilon_{1}$$

$$Model\;2:\;PSS=\alpha_{2}+\beta_{2}SNS+\left(covariates\right)+\varepsilon_{2},$$

where a binary variable of “SNS” is chosen from SNS_1 +_, SNS_10 +_, and SNS_100 +_, and *ɛ*_1_ and *ɛ*_2_ are error terms. Here, SNS is interpreted as a binary variable of SNS use corresponding to a given threshold of the number of SNS friends; if we choose SNS_10 +_, we consider the respondent as an SNS user only if they had 10 or more SNS friends.

In this SEM framework, we decomposed the effect of the number of SNS friends on LS into two components: not mediated by PSS (*β*_1_) and mediated by PSS (*β*_2_*γ*_1_). The total effect is the sum of these two components, *β*_1_ + *β*_2_*γ*_1_. After estimating Models 1 and 2 simultaneously, we calculated these components along with their standard errors as well as their proportions of the total effect of SNS use on LS. We conducted this SEM analysis for each SNS threshold as well as for each age group and compared the results.

To assess the robustness of the SEM analysis, we repeated a similar estimation, taking the number of SNS friends as a continuous variable. Considering the potential non-linear association between the number of SNS friends and LS, we compared the results obtained by using the entire sample and those obtained when excluding the respondents who had 100 or more SNS friends. For all statistical analyses, we used the software package Stata (Release 15) [27].

## Results

### Descriptive analysis

Key demographic features of the study sample are summarized in Table 1. No substantial differences were observed between samples collected in 2019 and 2020. Of the respondents, 43.9% graduated from college or above, 39.1% were regular employees, and 73.8% had a spouse.

**Table 1:** Key demographic features of the study sample.

Basic features		Survey year				Full sample	
		2019		2020			
		*n*	%	*n*	%	*n*	%
Gender							
	Men	5191	50.4	2670	50.6	7861	50.5
	Women	5102	49.6	2611	49.4	7713	49.5
Educational attainment							
	Junior high school	293	2.8	143	2.7	436	2.8
	High school	5487	53.3	2811	53.2	8298	53.3
	College or above	4513	43.8	2327	44.1	6840	43.9
Job status							
	Regular employee	3952	38.4	2140	40.5	6092	39.1
	Non-regular employee	2028	19.7	1109	21.0	3137	20.1
	Manager	190	1.8	113	2.1	303	1.9
	Self-employed	744	7.2	380	7.2	1124	7.2
	Doing a paid job at home	187	1.8	114	2.2	301	1.9
	Student with a job	537	5.2	299	5.7	836	5.4
	Student with no job	307	3.0	117	2.2	424	2.7
	Unemployed	347	3.4	139	2.6	486	3.1
	Out of labor force	2001	19.4	870	16.5	2871	18.4
Marital status							
	Having a spouse	7566	73.5	3924	74.3	11490	73.8
		*M*	*SD*	*M*	*SD*	*M*	*SD*
Age (years)		43.9	16.4	44.4	16.4	44.0	16.4
Household income		388.7	663.0	383.7	564.2	387.0	631.2
(annual, household-size-adjusted, 1000 JPY)							
*N*			10293		5281		15574

The distribution of the number of SNS friends between age groups is presented in Table 2. Having at least one SNS friend was more common among the young (83.9%) than the old (46.6%), and the proportion of SNS users who reportedly had 100 or more SNS friends was much higher among the young (32.0%) than the old (2.8%). One-way analysis of variance (ANOVA) conformed the variance between age groups for the number of SNS friends.

**Table 2:** Distribution of the number of SNS friends and one-way analyses of variance by age group.

Numbers of SNS friends	Young		Middle-aged		Old		All	
	*n*	%	*n*	%	*n*	%	*n*	%
None	588	16.1	2712	34.2	2121	53.4	5421	34.8
1–9	539	14.7	1804	22.7	960	24.2	3303	21.2
10–19	396	10.8	1012	12.7	427	10.7	1835	11.8
20–29	260	7.1	492	6.2	162	4.1	914	5.9
30–39	177	4.8	356	4.5	70	1.8	603	3.9
40–49	145	4.0	225	2.8	43	1.1	413	2.7
50–99	382	10.4	505	6.4	78	2.0	965	6.2
100–300	1052	28.8	761	9.6	89	2.2	1902	12.2
301+	120	3.3	74	0.9	24	0.6	218	1.4
ANOVA	*F*(2, 15574) 916.20***							
Total	3659	100.0	7941	100.0	3974	100.0	15574	100.0

^a^ Analysis of variance.

*** *p* < .001.

Table 3 presents how the distributions of LS and PSS were related to the number of SNS friends, by dividing the latter into four groups: none, 1–9, 10–99, and 100 or more. A larger number of SNS friends were generally correlated with higher levels of LS and PSS, and the one-way ANOVA confirmed the variance between levels of the number of SNS friends for LS and PSS. However, two issues should be noted. First, LS declined in response to an increase in SNS friends above 100 for the old, compared to additional increases for the young and middle-aged groups. Second, we observed a similar situation for PSS, which did not increase in response to an increase in SNS friends above 100 for the old. However, these results were not controlled for the covariates.

**Table 3:** Distribution of life satisfaction and perceived social support and one-way analyses of variance by the number of SNS friends.

Age group	Number of SNS friends	*n*	%	Life satisfaction^a^		Perceived social support^b^	
				*M*	*SD*	*M*	*SD*
Young	None	588	16.1	5.4	2.7	2.5	3.6
	1–9	539	14.7	5.4	2.4	2.6	2.8
	10–99	1360	37.2	5.9	2.1	3.7	3.9
	100 or more	1172	32.0	6.1	2.1	4.6	4.7
	Total	3659	100.0	5.8	2.3	3.6	4.1
	ANOVA^c^	*F*(3, 3659)		18.10***		51.38***	
Middle	None	2712	34.2	5.2	2.6	2.0	2.8
	1–9	1804	22.7	5.5	2.4	2.2	2.4
	10–99	2590	32.6	5.8	2.2	3.2	3.3
	100 or more	835	10.5	6.0	2.3	4.3	5.0
	Total	7941	100.0	5.5	2.4	2.7	3.3
	ANOVA	*F*(3, 7941)		43.99***		156.90***	
Old	None	2121	53.4	6.2	2.2	2.3	2.7
	1–9	960	24.2	6.4	2.1	2.5	2.4
	10–99	780	19.6	6.7	1.9	3.5	3.4
	100 or more	113	2.8	6.3	2.2	3.5	4.9
	Total	3974	100.0	6.3	2.1	2.6	2.9
	ANOVA	*F*(3, 3974)		12.38***		38.93***	

^a^ Ranging from 0 (*not satisfied at all*) to 10 (*highly satisfied*).

^b^ Indicates the number of persons who are expected to support them when needed (range: 0–40).

^c^Analysis of variance.

*** *p* < .001.

### Basic regression analysis

The estimation results of Model 0, which examined how the number of SNS friends was associated with LS and PSS, are summarized in Table 4, which contains the results after controlling for the covariates (Part A) and without controlling for them (Part B) for each of LSS and PSS. From the results for LS (Prat A), we noticed that the coefficients of SNS_10+_ (*β*_2_) were positive for all age groups, meaning that having 10 or more SNS friends was positively associated with LS. The coefficients of SNS_100+_ (*β*_3_) were non-significant for the young and middle-aged adults and turned even negative for the old. This suggests that an increase in SNS friends from already high levels did not increase or even reduce LS. Furthermore, the coefficient of SNS_1+_ (*β*_1_) was non-significant for the young, meaning that LS was not affected by an increase in SNS friends as long as it remained low. From the results for PSS (Part A), we also identified a positive association between SNS use and PSS for all age groups, as long as SNS users had 10 or more SNS friends. PSS was not affected by an increase in SNS friends above 100 for the old, unlike younger age groups whose PSS continued to rise. These results were generally consistent with the results of the descriptive analysis reported in Table 3.

**Table 4:** Regression analysis (Model 0): Estimated associations of the number of SNS friends with life-satisfaction and perceived social support^a^.

		Young			Middle–aged			Old		
		Estimate	*SE*	*p*	Estimate	*SE*	*p*	Estimate	*SE*	*p*
Dependent variable = life satisfaction^b^									
(A) Controlling for covariates										
	1 or more (*β*_01_)	.01	.13	.925	.22	.07	< .001	.25	.08	.003
	10 or more (*β*_02_)	.33	.11	.004	.23	.07	< .001	.20	.10	.043
	100 or more (*β*_03_)	.12	.09	.168	.06	.09	.505	–.53	.21	.013
	Women	.31	.08	< .001	.52	.06	< .001	.16	.07	.029
	High school	.07	.20	.723	.44	.16	.007	.21	.23	.355
	College or above	.23	.20	.251	.63	.16	< .001	.43	.23	.061
	Non–regular employee	–.27	.12	.024	–.24	.07	< .001	.08	.12	.534
	Manager	–.45	.33	.167	.30	.19	.115	.43	.22	.054
	Self–employed	–.22	.25	.383	–.04	.10	.662	.03	.14	.849
	Doing a paid job at home	–.45	.33	.171	–.25	.19	.194	.40	.23	.088
	Student with a job	.60	.11	< .001	.46	.61	.444	3.21	2.10	.126
	Student with no job	.43	.13	< .001	–.92	.80	.253	–2.18	1.48	.141
	Unemployed	–.91	.22	< .001	–.99	.14	< .001	–.32	.24	.177
	Out of labor force	–.03	.17	.843	.20	.09	.038	.41	.11	< .001
	Having a spouse	.40	.08	< .001	.89	.06	< .001	.44	.11	< .001
	Household income 2nd quartile	.35	.10	< .001	.28	.08	< .001	.54	.09	< .001
	Household income 3rd quartile	.45	.11	< .001	.65	.08	< .001	.69	.10	< .001
	Household income 4th quartile	.91	.11	< .001	1.16	.09	< .001	1.16	.11	< .001
	Age 30–39 years				.15	.07	.025			
	Age 40–49 years				.08	.07	.242			
	Age 50–59 years									
	Age 60 years +									
	Survey year	.09	.08	.273	.12	.05	.023	–.10	.07	.164
	Constant	4.48	.23	< .001	3.17	.18	< .001	4.66	.27	< .001
	Adjusted R^2^	.0622			.1074			.0523		
(B) Without controlling for covariates										
	1 or more (*β*_01_)	.08	.13	.567	.28	.07	< .001	.26	.08	.002
	10 or more (*β*_02_)	.42	.12	< .001	.38	.07	< .001	.27	.10	.010
	100 or more (*β*_03_)	.21	.09	.017	.11	.09	.228	–.38	.21	.077
	Constant	5.36	.09	< .001	5.18	.05	< .001	6.15	.05	< .001
	Adjusted R^2^	.0138			.0160			.0085		
Dependent variable = Perceived social support^c^									
(A) Controlling for covariates										
	1 or more (*β*_01_)	.03	.24	.907	.18	.10	.067	.20	.11	.071
	10 or more (*β*_02_)	1.05	.20	< .001	.88	.10	< .001	.96	.14	< .001
	100 or more (*β*_03_)	.85	.16	< .001	1.05	.13	< .001	–.06	.29	.836
	Women	–.29	.14	.032	.41	.08	< .001	.13	.10	.194
	High school	.75	.35	.033	.56	.23	.013	.39	.31	.204
	College or above	.87	.36	.016	.71	.23	.002	.44	.31	.157
	Non–regular employee	–.14	.22	.514	.08	.10	.467	.29	.16	.075
	Manager	.32	.58	.583	.82	.27	.002	.12	.30	.682
	Self–employed	.06	.45	.887	.46	.14	< .001	.44	.18	.017
	Doing a paid job at home	.52	.59	.380	–.23	.26	.373	.69	.32	.028
	Student with a job	.53	.19	.005	–1.08	.84	.199	21.22	2.83	< .001
	Student with no job	.17	.23	.474	.50	1.12	.657	–1.44	2.00	.471
	Unemployed	–.44	.40	.271	–.14	.20	.493	.95	.32	.003
	Out of labor force	–.51	.30	.091	–.14	.13	.299	.49	.15	.001
	Having a spouse	.50	.14	< .001	.41	.09	< .001	.15	.15	.338
	Household income 2nd quartile	.11	.19	.568	–.10	.11	.376	.37	.12	.001
	Household income 3rd quartile	–.01	.20	.949	.14	.12	.237	.57	.14	< .001
	Household income 4th quartile	.62	.19	< .001	.43	.12	< .001	.52	.15	< .001
	Age 30–39 years				.62	.09	< .001			
	Age 40–49 years				.27	.09	.005			
	Age 50–59 years									
	Age 60 years +									
	Survey year	.15	.14	.298	.15	.08	.050	.04	.09	.676
	Constant	1.44	.41	.000	.39	.25	.129	.94	.36	.010
	Adjusted R^2^	.0524			.0720			.047		
(B) Without controlling for covariates										
	1 or more (*β*_01_)	.10	.24	.660	.23	.10	.018	.22	.11	.043
	10 or more (*β*_02_)	1.08	.20	< .001	1.00	.10	< .001	.98	.14	< .001
	100 or more (_*β*03_)	.93	.16	< .001	1.12	.13	< .001	.06	.29	.823
	Constant	2.50	.16	< .001	1.99	.06	< .001	2.27	.06	< .001
	Adjusted R^2^	.040			.056			.028		
*n*		3659			7941			3974		

^a^ Controlled for covariates (gender, age group, educational attainment, job status, marital status, household income, and survey year).

^b^ Ranging from 0 (*not satisfied at all*) to 10 (*highly satisfied*).

^c^ Indicates the number of persons expected to support them when needed (range: 0–40).

In Table 4, we also observed that women tended to feel more satisfied with life and that educational attainment and household income were positively associated with LS and PSS. The table also contains the results without controlling for covariates (see Parts B). We confirmed that the control for the covariates did not increased the estimated coefficients of SNS use in most cases, suggesting the limited confounding effects of the covariates.

### Structural equation model analysis

We expanded the analysis to grasp a more comprehensive picture of the association among SNS, PSS, and LS using SEM analysis, through estimated Models 1 and 2. We estimated these models for each of the thresholds of SNS use (SNS_1+_, SNS_10+_, and SNS_100+_). Table 5 reports the estimation results of the major regression coefficients. This table indicates the positive associations between (1) LS and PSS (*γ*_1_ > 0) and (2) PSS and the number of SNS friends (*β*_2_ > 0) in all cases. The association between LS and the number of SNS friends, not mediated by PSS, was positive when using 1 or 10 SNS friends as the threshold (*β*_1_ > 0), but was non-significant when using 100 SNS friends as the threshold.

**Table 5:** Structural equation modeling analysis (Models 1 and 2): Estimated associations between the number of SNS friends, perceived social support, and life satisfaction.

Age group	Dependent	Explanatory variable	Number of SNS friends								
/ Model	variable	(coefficient)	1 or more			10 or more			100 or more		
			Estimate	*SE*	*p*	Estimate	SE	*p*	Estimate	SE	*p*
Young (*n* = 3659)											
Model 1	Life satisfaction	SNS friends (*β*_1_)	.20	.10	.05	.25	.08	< .001	.13	.08	.09
		Perceived social support (*γ*_1_)	.10	.01	< .001	.10	.01	< .001	.10	.01	< .001
Model 2	Perceived social support	SNS friends (*β*_2_)	1.18	.18	< .001	1.44	.15	< .001	1.31	.15	< .001
Log likelihood			–18287.6			–18258.0			–18268.6		
AIC			36653.1			36594.0			36615.2		
Middle-aged (*n* = 7941)											
Model 1	Life satisfaction	SNS friends (*β*_1_)	.26	.05	< .001	.22	.05	< .001	.07	.08	.44
		Perceived social support (*γ*_1_)	.12	.01	< .001	.12	.01	< .001	.13	.01	< .001
Model 2	Perceived social support	SNS friends (*β*_2_)	.88	.08	< .001	1.22	.07	< .001	1.62	.12	< .001
Log likelihood			–38081.0			–38015.5			–38065.0		
AIC			76248.1			76117.0			76215.9		
Old (*n* = 3974)											
Model 1	Life satisfaction	SNS friends (*β*_1_)	.24	.07	< .001	.18	.08	.02	–.33	.20	.10
		Perceived social support (*γ*_1_)	.11	.01	< .001	.11	.01	< .001	.12	.01	< .001
Model 3	Perceived social support	SNS friends (*β*_2_)	.65	.09	< .001	1.09	.11	< .001	.80	.27	< .001
Log likelihood			–18270.8			–18250.0			–18297.4		
AIC			36619.7			36577.9			36672.7		

^a^ Controlled for covariates (gender, age group, educational attainment, job status, marital status, household income, and survey year).

Based on these estimation results, we computed the effects of the number of SNS friends on LS, mediated and not mediated by PSS, as well as the proportion of these effects. From Table 6, which summarizes the results, we observed the following findings.

**Table 6:** Estimated effects of SNS use on life satisfaction, mediated and not mediated by perceived social support, based on Models 1 and 2.

Age group	Number of SNS friends								
Effect	1 or more			10 or more			100 or more		
	Estimate	*SE*	*P*	Estimate	*SE*	*p*	Estimate	*SE*	*p*
Young (*n* = 3659)									
Not mediated (*β*_1_)	.20	.10	.045	.25	.08	.002	.13	.08	.094
Mediated (*β*_2_*γ*_1_)	.12	.02	< .001	.14	.02	< .001	.13	.02	< .001
Total (*β*_1_ + *β*_2_*γ*_1_)	.32	.10	.002	.39	.08	< .001	.27	.08	< .001
Middle-aged (*n* = 7941)									
Not mediated (*β*_1_)	.26	.05	< .001	.22	.05	.000	.07	.08	.437
Mediated (*β*_2_*γ*_1_)	.11	.01	< .001	.15	.01	.000	.21	.02	< .001
Total (*β*_1_ + *β*_2_*γ*_1_)	.37	.05	< .001	.37	.05	.000	.27	.08	< .001
Old (*n* = 3974)									
Not mediated (*β*_1_)	.24	.07	< .001	.18	.08	.022	–.33	.20	.100
Mediated (*β*_2_*γ*_1_)	.07	.01	< .001	.12	.02	< .001	.09	.03	.005
Total (*β*_1_ + *β*_2_*γ*_1_)	.31	.07	< .001	.31	.08	< .001	–.23	.20	.248
Proportion of the effect mediated by perceived social support (*β*_2_*γ*_1_/[*β*_1_ + *β*_2_*γ*_1_]×100%)									
Young (*n* = 3659)	37.1	12.6	.003	36.5	8.6	< .001	49.4	15.7	.002
Middle-aged (*n* = 7941)	29.1	4.9	< .001	39.8	6.3	< .001	75.9	23.8	< .001
Old (*n* = 3974)	23.6	6.1	< .001	40.3	11.4	< .001			

First, LS generally had a positive association with SNS use, evidenced by the positive sign of the total association. For example, the estimated value of the total association of .39 (standard error [SE]: .08) for the young when using 10 SNS friends as the threshold for SNS use means that having 10 or more SNS friends is expected to raise LS by .39 compared to having less than 10 (including zero) SNS friends. The only exception for this positive association was the case using 100 SNS friends as the threshold for the old, which indicated a non-significant association. However, the association between SNS use and LS declined with a threshold higher than 100 SNS friends for the young and middle-aged groups. In addition, the magnitude of the positive association between SNS use and LS was somewhat smaller for the old than for the young and middle-aged groups.

Second, the table confirms the mediating effect of PSS on the association between SNS use and LS. This effect was positive and highly significant in all model specifications, and it was relatively stable for each age group. Notably, the estimated mediating effect for the old was .09 (SE: .03) for SNS_100+_, which lay in the range between .07 (SE: .01) and .12 (SE: .02) for lower thresholds, even though the total association between SNS use and LS turned non-significant for this age group.

Third, as seen in the bottom section of Table 6, the proportion of the effect mediated by PSS was generally substantial. For example, by taking 10 SNS friends as a threshold for SNS use, we found that the mediating effect of PSS explained 36.5% (SE: 8.6%), 39.8% (SE: 6.3%), and 40.3% (SE: 11.4%)of the total association between SNS use and LS for the young, middle-aged, and old groups, respectively. In the case of SNS_100+_ for the elderly, the proportion was minus 42.5%, meaning that it partly offset the overall negative association between SNS use and LS.

**Fig 1:**
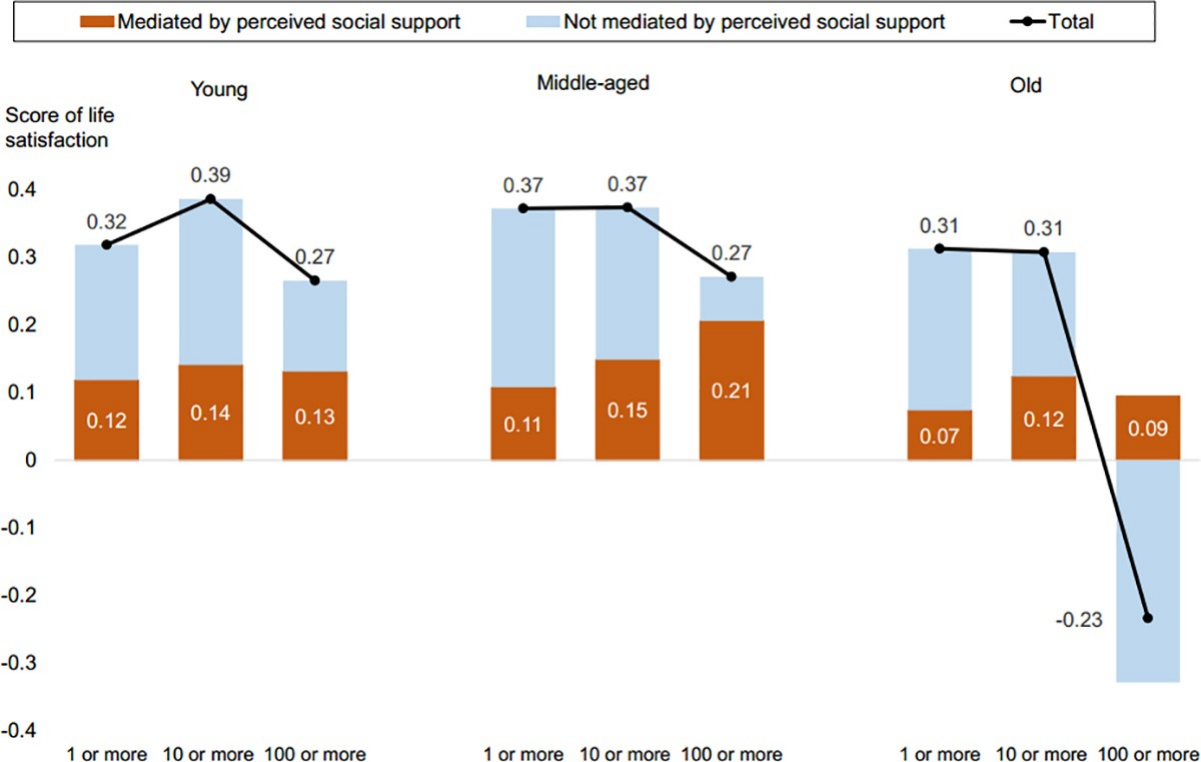
Association between the number of SNS friends and life satisfaction, mediated and not mediated by perceived social support.

To help understand the key estimation results, Fig 1 graphically illustrates how the mediating effect of PSS contributed to the total association between SNS use and LS for each combination of age group and threshold for SNS use. Based on the results in Table 6, this figure confirms the following: (1) there was a positive association between SNS use and LS, (2) it did not increase when the threshold was raised to 100 (and it actually turned negative for the old), and (3) the mediating effect of PSS consistently contributed to LS for all age groups and thresholds.

We further examined how the estimation results would be sensitive to the covariates. Tables 7 and 8 demonstrate how the results in Tables 5 and 6 changed if the covariates were not controlled for. We found that the general results of the mediation analysis remained intact, while the magnitude of the estimated associations between the variables was somewhat larger than that obtained after controlling for the covariates.

**Table 7:** Structural equation modeling analysis (Models 1 and 2): Estimated associations between the number of SNS friends, perceived social support, and life satisfaction [without controlling for covariates].

Age group	Dependent	Explanatory variable	The number of SNS friends								
/ Model	variable	(coefficient)	1 or more			10 or more			100 or more		
			Estimate	*SE*	*p*	Estimate	SE	*p*	Estimate	SE	*p*
Young (aged 15–29 years; *n* = 3659)											
Model 1	Life satisfaction	SNS friends (*β*_1_)	.36	.10	< .001	.39	.08	< .001	.26	.08	< .001
		Perceived social support (*γ*_1_)	.11	.01	< .001	.11	.01	< .001	.11	.01	< .001
Model 2	Perceived social support	SNS friends (*β*_2_)	1.35	.18	< .001	1.57	.14	< .001	1.44	.14	< .001
Log likelihood			–18420.8			–18384.0			–18398.2		
AIC			36855.5			36782.0			36810.5		
Middle–aged (aged 30–59 years; *n* = 7941)											
Model 1	Life satisfaction	SNS friends (*β*_1_)	.39	.06	< .001	.38	.05	< .001	.18	.09	0.04
		Perceived social support (*γ*_1_)	.15	.01	< .001	.14	.01	< .001	.15	.01	< .001
Model 2	Perceived social support	SNS friends (*β*_2_)	1.06	.08	< .001	1.41	.07	< .001	1.84	.12	< .001
Log likelihood			–38556.7			–38466.6			–38554.3		
AIC			77127.3			76947.2			77122.5		
Old (aged 60–86 years; *n* = 3974)											
Model 1	Life satisfaction	SNS friends (*β*_1_)	.28	.07	< .001	.26	.08	< .001	–.15	.20	0.45
		Perceived social support (*γ*_1_)	.12	.01	< .001	.12	.01	< .001	.13	.01	< .001
Model 3	Perceived social support	SNS friends (*β*_2_)	.70	.09	< .001	1.15	.11	< .001	.97	.27	< .001
Log likelihood			–18409.7			–18387.3			–18441.3		
AIC			36833.3			36788.6			36896.5		

**Table 8:** Estimated effects of SNS use on life satisfaction mediated and not mediated by perceived social support, based on Models 1 and 2 [without controlling for covariates].

Age group	The number of SNS friends								
Effect	1 or more			10 or more			100 or more		
	Estimate	*SE*	*p*	Estimate	*SE*	*p*	Estimate	*SE*	*p*
Young (*n* = 3659)									
Mediated (*β*_2_*γ*_1_)	.15	.02	< .001	.17	.02	< .001	.16	.02	< .001
Not mediated (*β*_1_)	.36	.10	< .001	.39	.08	< .001	.26	.08	< .001
Total (*β*_1_ + *β*_2_*γ*_1_)	.51	.10	< .001	.56	.08	< .001	.42	.08	< .001
Middle–aged (*n* = 7941)									
Mediated (*β*_2_*γ*_1_)	.16	.01	< .001	.20	.02	< .001	.28	.02	< .001
Not mediated (*β*_1_)	.39	.06	< .001	.38	.05	< .001	.18	.09	.035
Total (*β*_1_ + *β*_2_*γ*_1_)	.55	.06	< .001	.58	.05	< .001	.46	.09	< .001
Old (*n* = 3974)									
Mediated (*β*_2_*γ*_1_)	.09	.01	< .001	.14	.02	< .001	.13	.04	.001
Not mediated (*β*_1_)	.28	.07	< .001	.26	.08	.002	–.15	.20	.454
Total (*β*_1_ + *β*_2_*γ*_1_)	.37	.07	< .001	.40	.08	< .001	–.02	.20	.905
Proportion of the mediated effect (*β*_2_*γ*_1_/[*β*_1_ + *β*_2_*γ*_1_]×100%)									
Young (*n* = 3659)	30.0	7.0	< .001	30.3	5.3	< .001	38.0	8.0	< .001
Middle–aged (*n* = 7941)	28.3	3.6	< .001	35.0	4.0	< .001	60.5	11.7	< .001
Old (*n* = 3974)	23.7	5.4	< .001	35.6	8.2	< .001			

The estimation results obtained by replacing the binary variable of the number of SNS friends with their continuous variable are presented in Tables 9 and 10, corresponding to Tables 5 and 6, respectively. The results in Tables 9 and 10 are largely consistent with those in Tables 5 and 6. Regardless of whether respondents having 100 or more SNS friends were excluded, we confirmed an overall positive association between the number of SNS friends and LS as well as the mediating effect of PSS. Using the entire sample substantially reduced their magnitudes, especially for the old, probably reflecting a diminishing impact of an increasing number of SNS friends on LS, as observed in Tables 5 and 6.

**Table 9:** Structural equation modeling analysis (Models 1 and 2): Estimated associations between the number of SNS friends, perceived social support, and life satisfaction [using a continuous variable of the number of SNS friends]^a^.

	Dependent	Explanatory variable	Using the entire sample			Excluding respondents having 100 or more SNS friends		
	variable	(coefficient)	Estimate	*SE*	*p*	Estimate	SE	*p*
Young (*n* = 3659)								
Model 1	Life satisfaction	SNS friends (*β*_1_)	.0005	.0003	.146	.0030	.0018	.097
		Perceived social support (*γ*_1_)	.1002	.0090	< .001	.1209	.0123	< .001
Model 2	Perceived social support	SNS friends (*β*_2_)	.0061	.0006	< .001	.0179	.0029	< .001
Log likelihood			–18259.5			–12216.5		
AIC			36597.0			24511.0		
Middle–aged (*n* = 7941)								
Model 1	Life satisfaction	SNS friends (*β*_1_)	.0005	.0004	.184	.0034	.0014	.013
		Perceived social support (*γ*_1_)	.1257	.0079	< .001	.1385	.0092	< .001
Model 2	Perceived social support	SNS friends (*β*_2_)	.0083	.0005	< .001	.0202	.0017	< .001
Log likelihood			–38019.9			–33362.4		
AIC			76125.7			66810.8		
Old (*n* = 3974)								
Model 1	Life satisfaction	SNS friends (*β*_1_)	–.0003	.0007	.610	.0079	.0026	.002
		Perceived social support (*γ*_1_)	.1182	.0115	< .001	.1171	.0121	< .001
Model 2	Perceived social support	SNS friends (*β*_2_)	.0043	.0009	< .001	.0328	.0034	< .001
Log likelihood			–18291.9			–17628.6		
AIC			36661.7			35331.2		

^**a**^ Controlled for the covariates (gender, age group, educational attainment, job status, marital status, household income, and survey year).

**Table 10:** Estimated effects of SNS use on life satisfaction mediated and not mediated by perceived social support, based on Models 1 and 2 [using a continuous variable of the number of SNS friends].

	Using the entire sample			Excluding respondents having 100 or more SNS friends		
	Estimate	*SE*	*p*	Estimate	*SE*	*p*
Young (*n* = 3659)						
Mediated (*β*_2_*γ*_1_)	.0006	.0001	< .001	.0022	.0004	< .001
Not mediated (*β*_1_)	.0005	.0003	.146	.0030	.0018	.097
Total (*β*_1_ + *β*_2_*γ*_1_)	.0011	.0003	< .001	.0052	.0018	.005
Middle–aged (*n* = 7941)						
Mediated (*β*_2_*γ*_1_)	.0010	.0001	< .001	.0028	.0003	< .001
Not mediated (*β*_1_)	.0005	.0004	.184	.0034	.0014	.013
Total (*β*_1_ + *β*_2_*γ*_1_)	.0015	.0004	< .001	.0062	.0014	< .001
Old (*n* = 3974)						
Mediated (*β*_2_*γ*_1_)	.0005	.0001	< .001	.0038	.0006	< .001
Not mediated (*β*_1_)	–.0003	.0007	.610	.0079	.0026	.002
Total (*β*_1_ + *β*_2_*γ*_1_)	.0002	.0007	.806	.0117	.0026	< .001
Proportion of the mediated effect (*β*_2_*γ*_1_/[*β*_1_ + *β*_2_*γ*_1_]×100%)						
Young (*n* = 3659)	55.5	17.7	.002	41.9	15.7	< .001
Middle–aged (*n* = 7941)	68.8	16.5	< .001	45.3	10.5	< .001
Old (*n* = 3974)				32.8	8.3	< .001

## Discussion and conclusion

### Summary

This study aimed to compare the association between SNS use, PSS, and LS between age groups. Unlike most previous studies, we utilized a large-scale dataset obtained from a population-based, nationwide survey, covering a wide age range of the population. In addition to comparing how SNS was used between age groups in terms of the number of SNS friends, we examined how SNS use, PSS, and LS were associated with each other and how their associations differed between age groups. We observed that the number of SNS friends was positively associated with life satisfaction for all age groups and that the mediating effect of PSS mediated the association between the number of SNS friends and LS.

### Results and discussion

The key findings and their implications are summarized as follows. First, the results generally confirmed that SNS use was positively correlated with LS, in line with previous studies [7–9] that have argued that a higher number of SNS friends would enhance supportive interactions with others, subsequently having a positive impact on LS. More generally, SNS use may provide the opportunity to develop and maintain social connectedness in the online environment, which is associated with greater LS [20]. However, this association was found to be non-linear; if an SNS user already had many SNS friends, an increase in such friends was not associated with higher levels of LS, especially for the old. The association between SNS and SWB may depend on the level of SNS engagement, presumably leading to conflicting results. Having a substantially large number of SNS friends, along with frequent SNS use, may exacerbate the psychological pressure on SNS users, thereby offsetting the beneficial impact of SNS use to some extent.

Second, the results highlighted the importance of the mediating effect of PSS, as suggested in previous studies [6, 8, 23]. Our structural equation modeling analysis showed that PSS mediated a substantial portion of the positive association between SNS use and LS for all age groups, regardless of the number of SNS friends. Excluding this beneficial mediating effect of PSS, the association between SNS use and LS was non-significant for the young or middle-aged and even negative when they had many friends, making their overall association mixed.

Third, the results also showed that the association between SNS use and LS differed somewhat between age groups, as already shown by previous studies [12, 13], although they exhibited largely similar patterns in their association with respect to the number of SNS friends. More specifically, the sensitivity of LS to SNS use was somewhat more limited for the old compared to the young and middle-aged groups, and having 100 or more SNS friends was negatively associated with LS after excluding the association mediated by PSS. The results probably reflected differences in social contexts when using SNS as a tool of communication as well as exposure to digital technologies. Notably, younger people may use SNS as a means of immediate and social interaction more than the old [12], possibly making them more sensitive to the practical benefits of enhanced SNS use.

### Theoretical implications

The results underscored the mediating effect of PSS on the positive association between SNS use and LS, as hypothesized. Notably, this mediating effect was consistently observed across age groups, regardless of the number of SNS friends, and it accounted for a substantial proportion of the association between SNS use and LS. This result suggests that all age groups have two pathways linking SNS use to LS: (1) a larger number of SNS friends makes SNS users perceive more social support from others, and (2) an enhanced perception of social support adds to life satisfaction [6, 8, 23]. These two pathways appear to work effectively even if an SNS user already has many SNS friends. However, more in-depth analysis of the PSS’ mediating effect is needed, considering that previous studies argued for its non-linearity, dependency on personal traits, and limitations to some of its aspects [23–25].

### Practical implications

The results suggest that SNS use has the potential to improve SWB via its positive impact on PSS. Policy measures to promote SNS use can be expected to enhance social welfare. More specifically, policy support to encourage individuals who have some difficulties in receiving social support to use SNS is expected to have a beneficial impact on their LS. It is also advisable for policymakers to provide more technical support for SNS use to the old, who are likely to be less familiar with SNS technology than the young and middle-aged.

### Limitations

We recognize that this study has several limitations in addition to the potential measurement errors and biases in the self-reported measures of the number of SNS friends, the number of persons expected to provide support, and life satisfaction. First, the cross-sectional data setting could not identify causation. Notably, the reverse causality from LS to SNS use or PSS cannot be excluded. Equally important, an individual’s unobserved attributes, including personality traits, may have caused biased estimation results, as already suggested by preceding studies [28, 29]. To control for potential biases, we must expand the analysis using longitudinal data.

Second, and related to the above, analysis should be expanded to the dynamic association between SNS use and SWB. In this cross-sectional study, we could not analyze how each individual’s SWB evolved in response to a change in SNS use. SWB may likely change over time, and the intensity of SNS use, which was not considered in this study, may play a key role in the evolution of SWB [30]. The feedback loop from SWB to SNS use should also be examined in these dynamics.

Third, while we focused on PSS, there can be another potential mediator that links SNS use to SWB. The chance of obtaining instrumental social support as well as perceived support may be enhanced by SNS use, and the formation and maintenance of social capital can also be promoted by online communications [31]. In addition, the mediating effect of possible behavioral modifications in daily life triggered by SNS use should be considered to fully capture its impact on SWB.

Despite these limitations, this study showed that the mediating effect of PSS contributed to the positive association between SNS use and LS regardless of the number of SNS friends. We consistently observed this effect for all age groups, although the association between SNS use and LS differed somewhat between the young and old groups. These results suggest that SNS use has the potential to enhance SWB via its positive impact on PSS.
